# Encounters and medication use for ocular surface disorders among patients treated with dupilumab: A cohort study

**DOI:** 10.1016/j.jdin.2021.03.009

**Published:** 2021-04-29

**Authors:** John S. Barbieri, Vatinee Y. Bunya, Mina Massaro-Giordano, David J. Margolis

**Affiliations:** a Department of Dermatology, University of Pennsylvania Perelman School of Medicine, Philadelphia, Pennsylvania; b Department of Ophthalmology, University of Pennsylvania Perelman School of Medicine, Philadelphia, Pennsylvania; c Center for Clinical Epidemiology and Biostatistics, Department of Biostatistics, Epidemiology and Informatics, University of Pennsylvania Perelman School of Medicine, Philadelphia, Pennsylvania

**Keywords:** conjunctivitis, dry eye, dupilumab, general dermatology, medical dermatology, ocular surface disorders, ophthalmology

## Abstract

**Background:**

Although dupilumab has been associated with the development of conjunctivitis, little is known about other ocular surface disorders such as dry eye and how these side effects are managed.

**Objective:**

To evaluate the incidence and management of ocular surface disorders, including dry eye and conjunctivitis, among patients treated with dupilumab.

**Methods:**

Using US claims data, we evaluated the incidence of encounters for ocular surface disorders among patients treated with dupilumab. Secondary outcomes included ophthalmic medication use. A propensity score matched, active-comparator, new-user cohort design was used to compare the incidence of ocular surface disorders between those starting dupilumab versus methotrexate.

**Results:**

Among those with a history of atopic dermatitis, encounters for ocular surface disorders were more common in the 6 months after starting dupilumab than in the 6 months prior (11.7% versus 8.7%, *P* < .001); 59.7% of those with a new ocular surface disorder diagnosis filled a prescription for an ophthalmic medication. The incidence of ocular surface disorders was higher among those treated with dupilumab than that in those treated with methotrexate (odds ratio 1.64; 95% confidence interval 1.17-2.30).

**Limitations:**

Observational design.

**Conclusions:**

Dupilumab use for atopic dermatitis was associated with an increased risk of ocular surface disorders. Most patients who developed an ocular surface disorder received a prescription for an ophthalmic medication.

**Capsule Summary**

• Dupilumab was associated with an increased risk of ocular surface disorders such as dry eye and conjunctivitis among those treated for atopic dermatitis but not among those treated for asthma.
• Given frequent use of ophthalmic medications in this population, early referral to ophthalmology could be considered.

## Introduction

Dupilumab is a monoclonal antibody targeting interleukin 4 and interleukin 13 signaling that is approved for atopic dermatitis, asthma, and chronic rhinosinusitis with nasal polyps.^1^ Although it was found to be effective for these indications, conjunctivitis occurred in 8.6%-22.1% of those treated with dupilumab for atopic dermatitis compared with 2.1%-11.1% of those who received placebo.^2^, ^3^, ^4^, ^5^ Higher baseline severity of atopic dermatitis and history of conjunctivitis were associated with an increased risk of developing conjunctivitis while on dupilumab. A cohort study also identified an association between dupilumab use for atopic dermatitis and conjunctivitis; however, it did not evaluate other ocular surface disorders such as dry eye or clinical management.^6^ Several small case series have also described ocular surface disorders among 10%-40% of patients being treated with dupilumab.^7^, ^8^, ^9^, ^10^ In contrast, in the phase III trials of dupilumab for asthma and chronic rhinosinusitis with nasal polyps, an increased risk for conjunctivitis was not observed.^2^^,^^11^^,^^12^

Although these studies highlight the importance of ocular surface disorders as potential complications of dupilumab use, they have several important weaknesses including small sample size, short duration of follow-up, and limited generalizability to routine clinical practice scenarios. In addition, most of these studies focused on conjunctivitis without considering other ocular surface disorders that may be associated with dupilumab such as dry eye, and few examined treatment use.^2^^,^^6^^,^^7^^,^^11^^,^^12^ The purpose of this study was to comprehensively evaluate the incidence of ocular surface disorders (ie, conjunctivitis and dry eye) among a large, population-based cohort of patients being treated with dupilumab in routine clinical practice, as well as to examine the use of ophthalmic treatments and the frequency of dupilumab discontinuation.

## Materials and methods

### Data source

This retrospective cohort study used the Optum de-identified database (Clinformatics Data Mart Database). The Optum database includes de-identified commercial claims data for approximately 12-14 million annual covered individuals in the United States. These data include both medical and pharmacy claims, as well as patient demographic information such as age and sex. The patient population available in the Optum database is similar to the demographics of the United States population with respect to sex, age, and geographic distribution.^13^

### Study population and outcomes

The study period was between January 1, 2016, and June 30, 2019. Study inclusion criteria were: at least 6 months of continuous enrollment prior to and after the index date, which was defined as the date of the first prescription for dupilumab. International Classification of Diseases 10^th^ revision codes were used to classify patients as having a history of atopic dermatitis (L20.8x, L20.9), asthma (J45x), or seasonal allergies (J30.1, J30.2, J30.8, J30.9), which were assessed during the 6 months prior to the index date.

The primary outcome was the incidence of encounters for an ocular surface disorder, which was defined as any encounter with an International Classification of Diseases 10th revision code for dry eye syndrome, atopic conjunctivitis, or other conjunctivitis in the 6 months prior to and the 6 months after the index date (Table I). To examine the impact of the development of ocular surface disorders on treatment, the frequency of dupilumab discontinuation within 45 days of the encounter for an ocular surface disorder was evaluated.

**Table I:** International Classification of Diseases 10th revision codes used to classify dry eye, atopic conjunctivitis, and other conjunctivitis

Description	Categorization	ICD-10 Code
Dry eye syndrome	Dry eye	H04.123
Keratoconjunctivitis sicca, not Sjogren syndrome	Dry eye	H16.223
Tear film insufficiency	Dry eye	H04.129
Blepharoconjunctivitis	Dry eye	H10.509
Blepharitis	Dry eye	H01.0
Blepharitis of both eyes	Dry eye	H01.003
		H01.006
Blepharitis, unspecified eye, unspecified eyelid	Dry eye	H01.009
Blepharitis of eyelid of both upper and lower portion of left eye	Dry eye	H01.00B
Blepharitis of eyelid of both upper and lower portion of right eye	Dry eye	H01.00A
Blepharitis of lower portion of left eyelid	Dry eye	H01.005
Blepharitis of lower portion of right eyelid	Dry eye	H01.002
Blepharitis of upper portion of right lid	Dry eye	H01.001
Blepharitis of upper portion left lid	Dry eye	H01.004
Meibomian gland dysfunction	Dry eye	H02.889
Meibomian gland dysfunction of both eyes	Dry eye	H02.883
		H02.886
Meibomian gland dysfunction of lower portion of left eyelid	Dry eye	H02.885
Meibomian gland dysfunction of upper portion of left eyelid	Dry eye	H02.884
Meibomian gland dysfunction of eyelids of upper and lower portion of left eye	Dry eye	H02.88B
Meibomian gland dysfunction of eyelids of lower portion of right eye	Dry eye	H02.882
Meibomian gland dysfunction of upper portion of right eyelid	Dry eye	H02.881
Meibomian gland dysfunction of eyelids of upper and lower portion of right eye	Dry eye	H02.88A
Dry eye syndrome of right eye due to meibomian gland dysfunction	Dry eye	H04.121
		H02.883
Dry eye syndrome of left eye due to meibomian gland dysfunction	Dry eye	H04.122
		H02.886
Acute atopic conjunctivitis	Atopic conjunctivitis	H10.10
Allergic conjunctivitis	Atopic conjunctivitis	H10.13
Other chronic allergic conjunctivitis	Atopic conjunctivitis	H10.45
Acute conjunctivitis	Other conjunctivitis	H10.30
Chronic conjunctivitis	Other conjunctivitis	H10.409
Conjunctivitis	Other conjunctivitis	H10.9
Red eye/redness/discharge of eye	Other conjunctivitis	H57.89

*ICD-10*, International Classification of Diseases, 10^th^ revision.

To examine the utilization patterns of ophthalmic medications, we assessed the frequency of filled pharmacy claims for ophthalmic steroids, antibiotics, steroid–antibiotic combination products, nonsteroidal anti-inflammatory drugs, cyclosporine, and lifitegrast in the 6 months prior to and the 6 months after the index date.

In addition, to better understand the relative influence of dupilumab on the incidence of ocular surface disorders, among those with a history of atopic dermatitis and no history of ocular surface disorders, we used an active-comparator, new-user design to compare the incidence of encounters for ocular surface disorders between those with atopic dermatitis starting dupilumab and those starting methotrexate.

### Statistical analyses

Differences in the incidence of ocular surface disorders and ophthalmic medication use in the 6 months before and after starting dupilumab were compared using paired *t* tests. Because differences in the incidence of ocular surface orders between those with atopic dermatitis and those with asthma were observed in the clinical trials, we stratified our results by those with a history of atopic dermatitis (with or without a history of asthma) and those with a history of asthma but not atopic dermatitis. In addition, we performed sensitivity analyses in which we only evaluated those with no history of ocular surface disorders in the 6 months prior to the index date.

For the active-comparator, new-user comparison of the incidence of ocular surface disorders among those with atopic dermatitis and no history of ocular surface disorders, we used propensity score matching on sex, history of asthma, history of seasonal allergies, and age at the index date. These variables were chosen to reduce the potential selection bias between the cohorts. The propensity scores were used to create 5 quintiles. Multivariate logistic regression and linear regression, adjusting for sex, history of asthma, history of seasonal allergies, and age at the index date, were used to compare the odds of the development of any ocular surface disorder in the 6 months after the index date for each quintile. An overall analysis was performed by including quintile in the model. Statistical analyses were performed using Stata 16 (StataCorp). This study was deemed exempt by the Institutional Review Board of the University of Pennsylvania and conducted in adherence with the STROBE and RECORD guidelines.^14^

## Results

There were 2144 patients identified with a prescription for dupilumab during the study period, of whom 1816 (84.7%) had a history of atopic dermatitis and 129 (6.0%) had a history of asthma without a history of atopic dermatitis. Among the atopic dermatitis cohort, the mean age was 47.4 years (Standard deviation 18.5) and 51.9% were female; 13.9% had a history of asthma alone, 16.6% had a history of seasonal allergies alone, and 16.4% had a history of both asthma and seasonal allergies (Table II).

**Table II:** Patient characteristics

	History of atopic dermatitis	History of asthma only	All patients
No.	1816	129	2144
History of asthma, N (%)	253 (13.9)	-	299 (14.0)
History of seasonal allergies, N (%)	302 (16.6)	83 (64.3)	330 (15.4)
History of asthma and allergies, N (%)	298 (16.4)	-	381 (17.8)
Female, N (%)	942 (51.9)	66 (51.2)	1106 (51.6)
Course duration, days, mean (SD)	340 (196)	298 (187)	334 (196)
Course duration, days, median (IQR)	309 (208-487)	247 (184-421)	303 (203-479)
Age at first prescription, years, mean (SD)	47.4 (18.5)	46.6 (19.6)	48.0 (18.7)

*IQR*, Interquartile range; *SD*, standard deviation.

Among those with a history of atopic dermatitis, 8.7% had an encounter for an ocular surface disorder in the 6 months prior to starting dupilumab compared to 11.7% in the 6 months after starting dupilumab (*P* = .0007); among those with no prior history of an ocular surface disorder, 9.6% had an encounter for an ocular surface disorder in the 6 months after starting dupilumab. Among those with no prior history of an ocular surface disorder, the median time from starting dupilumab to this encounter was 76 (interquartile range: 49-124) days. Dupilumab was discontinued for any reason within 45 days of this encounter by 13.8% of patients. If restricted to encounters with an ophthalmologist, 3.1% had an encounter for an ocular surface disorder in the 6 months prior to starting dupilumab compared with 4.5% in the 6 months after starting dupilumab (*P* = .02). There were relatively similar proportion of patients diagnosed with conjunctivitis and dry eye (Table III).

**Table III:** Encounters for ocular surface disorders

	History of atopic dermatitis	History of asthma only	All patients
Encounter for ocular surface disorder before starting dupilumab, n (%)	158 (8.7)	22 (17.1)	201 (9.4)
Conjunctivitis			
Atopic conjunctivitis (H10.1x), n (%)	75 (4.1)	14 (10.9)	99 (4.6)
All other conjunctivitis, n (%)	5 (0.3)	0 (0)	7 (0.3)
Both, n (%)	3 (0.2)	0 (0)	3 (0.1)
Dry eye, n (%)	86 (4.7)	8 (6.2)	103 (4.8)
Encounter for ocular surface disorder after starting dupilumab, n (%)	213 (11.7)	24 (18.6)	255 (11.9)
Conjunctivitis			
Atopic conjunctivitis (H10.1x), n (%)	79 (4.4)	9 (7.0)	97 (4.5)
All other conjunctivitis, n (%)	35 (1.9)	1 (0.8)	38 (1.8)
Both, n (%)	6 (0.3)	0 (0)	7 (0.3)
Dry eye, n (%)	111 (6.1)	16 (12.4)	133 (6.2)
Time from start of dupilumab to encounter for ocular surface disorder, days, median (IQR)	71 (44-121)	62 (20-91)	71 (42-116)
Discontinued dupilumab within 45 days of encounter, n (%)	31 (14.6)	2 (8.3)	36 (14.1)
Encounter for ocular surface disorder after starting dupilumab among those without a history of ocular surface disorder in the 6 months prior to starting dupilumab, n (%)	159 (9.6)	14 (13.1)	137 (7.9)
Conjunctivitis			
Atopic conjunctivitis (H10.1x), n (%)	44 (2.4)	5 (3.9)	51 (2.4)
All other conjunctivitis, n (%)	33 (1.8)	1 (0.8)	36 (1.7)
Both, n (%)	5 (0.3)	0 (0)	5 (0.2)
Dry eye, n (%)	88 (5.3)	9 (8.4)	101 (5.2)
Time from start of dupilumab to encounter for ocular surface disorder, days, median (IQR)	76 (49-124)	62 (23-92)	74 (49-120)
Discontinued dupilumab within 45 days of encounter, n (%)	22 (13.8)	2 (14.3)	26 (14.4)
Encounter for ocular surface disorder with an ophthalmologist before starting dupilumab, n (%)	57 (3.1)	4 (3.1)	66 (3.1)
Conjunctivitis			
Atopic conjunctivitis (H10.1x), n (%)	13 (0.7)	1 (0.8)	14 (0.7)
All other conjunctivitis, n (%)	0 (0)	0 (0)	0 (0)
Both, n (%)	0 (0)	0 (0)	0 (0)
Dry eye, n (%)	45 (2.5)	3 (2.3)	53 (2.5)
Encounter for ocular surface disorder with an ophthalmologist after starting dupilumab, n (%)	81 (4.5)	12 (9.3)	98 (4.6)
Conjunctivitis			
Atopic conjunctivitis (H10.1x), n (%)	19 (1.0)	2 (1.6)	22 (1)
All other conjunctivitis, n (%)	2 (0.1)	0 (0)	2 (0.1)
Both, n (%)	0 (0)	0 (0)	0 (0)
Dry eye, n (%)	63 (3.5)	10 (7.8)	77 (3.6)
Encounter for ocular surface disorder with an ophthalmologist after starting dupilumab among those without a history of ocular surface disorder in the 6 months prior to starting dupilumab, n (%)	67 (3.8)	9 (7.2)	79 (3.8)
Conjunctivitis			
Atopic conjunctivitis (H10.1x), n (%)	18 (1.0)	2 (1.6)	21 (1)
All other conjunctivitis, n (%)	2 (0.1)	0 (0)	2 (0.1)
Both, n (%)	0 (0)	0 (0)	0 (0)
Dry eye, n (%)	50 (2.8)	7 (5.6)	59 (2.8)

*IQR*, Interquartile range.

Among those with a history of asthma and no history of atopic dermatitis, 17.1% had an encounter for an ocular surface disorder in the 6 months prior to starting dupilumab compared with 18.6% in the 6 months after starting dupilumab (*P* = .70); among those with no prior history of an ocular surface disorder, 13.1% had an encounter for an ocular surface disorder in the 6 months after starting dupilumab. Among those with no prior history of an ocular surface disorder, the median time from the start of dupilumab to this encounter was 62 (interquartile range: 23-92) days (Table III).

In the active-comparator, new-user analysis, there was adequate covariate balance achieved within each quintile (Table IV). Among those with a history of atopic dermatitis and no history of ocular surface disorders, those treated with dupilumab were more likely to have an encounter for an ocular surface disorder after starting treatment than those treated with methotrexate (odds ration 1.64; 95% confidence interval: 1.17-2.30). The absolute incidence of encounters for ocular surface disorders was 3.3% (95% confidence interval: 1.0%-5.7%) higher among those treated with dupilumab (Table V).

**Table IV:** Covariate balance within quintiles for propensity score analysis

Quintile	N		History of asthma, %		History of seasonal allergy, %		Female, %		Age, years, mean	
	Dupilumab	MTX	Dupilumab	MTX	Dupilumab	MTX	Dupilumab	MTX	Dupilumab	MTX
1	162	338	2.5%	2.7%	11.1%	9.2%	72.2%	81.7%	73.0	74.6
2	221	266	8.6%	12.0%	18.1%	17.7%	63.8%	49.6%	61.5	65.1
3	288	204	14.9%	20.1%	22.2%	23.0%	51.4%	51.0%	52.4	53.6
4	368	119	29.4%	29.4%	28.3%	29.4%	49.7%	52.1%	40.2	41.3
5	382	108	51.8%	35.2%	55.8%	46.3%	37.2%	46.3%	27.3	18.0
Overall	1421	1035	26.2%	15.0%	30.9%	20.3%	51.4%	60.3%	46.3	58.3

*MTX*, Methotrexate.

**Table V:** Active-comparator, new-user propensity score analysis

Quintile	OR, adj∗	% diff, adj†
1	0.57 (0.26 to 1.25)	−3.7 (−8.9 to 1.6)
2	2.07 (1.04 to 4.15)	5.2 (0.3 to 10.1)
3	1.38 (0.60 to 3.17)	1.6 (−2.5 to 5.6)
4	7.18 (1.70 to 30.41)	9.2 (3.4 to 15.0)
5	1.88 (0.70 to 5.05)	3.3 (−3.5 to 10.2)
Overall	1.64 (1.17 to 2.30)	3.3 (1.0 to 5.7)

*% diff*, Absolute percent difference; *adj*, adjusted; *OR*, odds ratio.

∗ Adjusted for sex, history of asthma, history of seasonal allergies, and age at the index date.

† adjusted odds ratio and percent difference are for methotrexate compared with dupilumab.

Among those with a history of atopic dermatitis, 9.4% filled a prescription for an ophthalmic medication in the 6 months prior to starting dupilumab compared with 13.8% in the 6 months after starting dupilumab (*P* = .0001); among those who developed a new diagnosis of an ocular surface disorder, 59.7% filled a prescription for an ophthalmic medication in the 6 months after starting dupilumab. The most commonly prescribed medication classes were topical steroid (27.0%), combination topical steroid–antibiotic (17.5%), topical antibiotic (17.0%), and topical antihistamine (14.5%) drops. Among those who saw an ophthalmologist, there was higher use of topical steroid (41.8% versus 20.2%, *P* = .003), cyclosporine (7.5% versus 4.0%, *P* = .34), and lifitegrast drops (3.0% versus 2.0%, *P* = .69) (Table VI).

**Table VI:** Ophthalmic medication use

	History of atopic dermatitis	History of asthma only	All patients
	n (%)	n (%)	n (%)
Any ophthalmic medication use prior to starting dupilumab	170 (9.4)	22 (17.1)	204 (9.5)
Topical antihistamines	52 (2.9)	4 (3.1)	58 (2.7)
Topical steroids	64 (3.5)	10 (7.8)	77 (3.6)
Topical steroid antibiotics	25 (1.4)	2 (1.6)	31 (1.4)
Topical antibiotics	71 (3.9)	8 (6.2)	83 (3.9)
Topical NSAIDs	13 (0.7)	0 (0)	14 (0.7)
Topical cyclosporine	11 (0.6)	1 (0.8)	13 (0.6)
Topical lifitegrast	7 (0.4)	1 (0.8)	9 (0.4)
Any ophthalmic medication use after starting dupilumab	251 (13.8)	24 (18.6)	300 (14)
Topical antihistamines	64 (3.5)	6 (4.7)	78 (3.6)
Topical steroids	103 (5.7)	7 (5.4)	117 (5.5)
Topical steroid antibiotics	67 (3.7)	4 (3.1)	78 (3.6)
Topical antibiotics	77 (4.2)	10 (7.8)	96 (4.5)
Topical NSAIDs	16 (0.9)	0 (0)	17 (0.8)
Topical cyclosporine	18 (1.0)	2 (1.6)	21 (1.0)
Topical lifitegrast	12 (0.7)	2 (1.6)	16 (0.7)
Any ophthalmic medication use after starting dupilumab, among those who developed a new ocular surface disorder diagnosis	95 (59.7)	6 (42.9)	103 (56.9)
Topical antihistamines	23 (14.5)	1 (7.1)	25 (13.8)
Topical steroids	43 (27.0)	0 (0)	44 (24.3)
Topical steroid antibiotics	28 (17.6)	1 (7.1)	30 (16.6)
Topical antibiotics	27 (17.0)	2 (14.3)	30 (16.6)
Topical NSAIDs	3 (1.9)	0 (0)	3 (1.7)
Topical cyclosporine	9 (5.7)	0 (0)	9 (5.0)
Topical lifitegrast	3 (1.9)	2 (14.3)	5 (2.8)
Any ophthalmic medication use after starting dupilumab, among those who developed a new ocular surface disorder diagnosed by an ophthalmologist	44 (65.7)	6 (66.7)	51 (64.6)
Topical antihistamines	13 (19.4)	1 (11.1)	14 (17.7)
Topical steroids	28 (41.8)	2 (22.2)	31 (39.2)
Topical steroid antibiotics	13 (19.4)	1 (11.1)	14 (17.7)
Topical antibiotics	15 (22.4)	3 (33.3)	19 (24.1)
Topical NSAIDs	4 (6.0)	0 (0)	4 (5.1)
Topical cyclosporine	5 (7.5)	0 (0)	5 (6.3)
Topical lifitegrast	2 (3.0)	1 (11.1)	3 (3.8)

*NSAIDs*, Nonsteroidal anti-inflammatory drugs.

Among those with a history of asthma, 17.1% filled a prescription for an ophthalmic medication in the 6 months prior to starting dupilumab compared with 18.6% in the 6 months after starting dupilumab (*P* = .70); among those who developed a new diagnosis of an ocular surface disorder, 42.9% filled a prescription for an ophthalmic medication in the 6 months after starting dupilumab (Table VI).

## Discussion

Consistent with prior trials, our findings highlighted that the use of dupilumab in patients with atopic dermatitis was associated with a significant increased incidence of encounters for ocular surface disorders.^2^, ^3^, ^4^, ^5^ There appeared to be an increase in both the incidence of encounters for dry eye as well as encounters for nonatopic conjunctivitis. Over 85% of patients with an encounter for an ocular surface disorder after starting dupilumab did not discontinue the medication within 45 days of this encounter. These findings support that in routine clinical practice, ocular surface disorders significant enough to seek medical attention are relatively common but often can be managed to allow for continued treatment.

Consistent with prior studies, we identified that the onset of encounters for ocular surface disorders may occur sometime after the initiation of dupilumab.^2^^,^^7^ The median time from the start of dupilumab to the first encounter for a diagnosis of a new ocular surface disorder was approximately 11 weeks in our study. This finding highlights the importance of remaining vigilant for the development of ocular surface disorders in patients being treated with dupilumab, as this side effect may not become apparent until later in the course of treatment.

Nearly 60% of patients who had an encounter for a new ocular surface disorder after starting dupilumab received a prescription for an ophthalmic medication. Although evidence is limited regarding the optimal prevention and management of ocular surface disorders in the setting of dupilumab treatment, topical steroids, tacrolimus, cyclosporine, and lifitegrast drops have been suggested for the management of moderate-to-severe conjunctivitis.^15^ In our study, the most commonly prescribed medications were topical steroids, topical antibiotics, and topical antihistamines. In particular, the frequent use of topical antibiotics may not be aligned with optimal management of dupilumab-associated ocular surface disorders. Cyclosporine and lifitegrast drops were rarely prescribed, although use of these medications and topical steroid drops was more common among those seen by an ophthalmologist. Because topical steroid drops can have ocular side effects such as cataracts and glaucoma, patients should be monitored by an ophthalmologist while receiving these medications.^16^

Given the high prevalence of ocular surface disorders at baseline and the increased risk of ocular surface disorders with dupilumab treatment, it may be reasonable to consider early ophthalmology referral prior to starting dupilumab or at the first sign of ocular symptoms. A small study of 25 patients found that 64% had an abnormal ocular surface examination at baseline prior to starting dupilumab.^17^ Two small nonrandomized studies suggested that prophylactic use of artificial tears can reduce the incidence of ocular surface disorders among those being treated with dupilumab.^17^^,^^18^ Initiating artificial tears with dupilumab is a relatively simple approach that could be considered for patients with atopic dermatitis planning to initiate treatment with dupilumab.^19^

In contrast to the clinical trials for asthma, we identified a high incidence of ocular surface disorders both before and after starting dupilumab among our study cohort with a history of asthma and no history of atopic dermatitis. There are several potential explanations for this finding. Nearly two-thirds of the individuals in this cohort had a history of seasonal allergies, which could correlate with the relatively high incidence of atopic conjunctivitis compared with dry eye and other conjunctivitis observed in our study. However, in the VENTURE trial 56.7% of subjects had a history of allergic rhinitis and in the QUEST trial 87.7% of subjects had an ongoing atopic or allergic condition at baseline, making this explanation less likely, ^11^^,^^12^ although it is possible that conjunctivitis in the setting of treatment with dupilumab was rarely reported in these studies given the high prevalence of allergic conditions at baseline. Another possibility is that the trial investigators may have had a higher threshold to document mild ocular surface disorders than clinicians in routine clinical practice. Importantly, similar to the clinical trials, we did not identify a significant difference in the incidence of ocular surface disorders or ophthalmic medication use in the 6 months before and after starting dupilumab.^2^ These findings support that the risk of dupilumab-associated ocular surface disorders may be lower among patients treated for asthma.

This study has several strengths, including the large sample size with >2000 patients treated with dupilumab, which is substantially larger than that of previous studies on this topic. The use of data collected in routine clinical practice increases the generalizability of the findings. In addition, by comprehensively evaluating ocular surface disorders, including dry eye, and by attempting to differentiate atopic and other forms of conjunctivitis, our study provides a more granular analysis of ocular surface disorders compared with those of prior cohort studies.^2^^,^^6^

The findings of this study should be interpreted in the context of the study design. because concerns regarding dupilumab-associated ocular surface disorders have been present since the initial clinical trials, it is possible that clinicians would be more likely to evaluate for and code ocular surface disorders among patients receiving dupilumab than among those receiving methotrexate. In addition, there is the possibility for misclassification bias given the use of International Classification of Diseases 10th revision codes, and we are unable to determine whether patients with encounters for ocular surface disorders were thought to have developed this condition because of dupilumab. However, the use of an active-comparator, new-user design, and conducting the sensitivity analyses among those with no prior history of ocular surface disorders mitigates this risk to some degree. Because we were unable to differentiate cutaneous versus ophthalmic use of topical calcineurin inhibitors, we were not able to evaluate the use of topical calcineurin inhibitors for ocular surface disorders. Although our overall study population was large, we have smaller sample sizes for patients treated with dupilumab who have a history of asthma only and we have a limited sample size for some of the less commonly prescribed ophthalmic medications; future studies with larger populations are needed to confirm our findings.

## Conclusions

Ocular surface disorders were common among patients with atopic dermatitis and asthma being treated with dupilumab. In addition, treatment with dupilumab was associated with a significant increased risk of ocular surface disorders among those with atopic dermatitis but not asthma. Most patients who developed an ocular surface disorder received a prescription for an ophthalmic medication. Early referral to ophthalmology in anticipation of starting dupilumab or at the development of ocular symptoms may have the potential to improve prevention and management of ocular surface disorders, although future prospective studies are needed.

## Conflicts of interest

Dr Margolis was a member of joint Sanofi/Regeneron Data Safety and Monitoring Board for studies of dupilumab; he left the Data Safety and Monitoring Board about 2 years ago. He receives research funding to the Trustees of the University of Pennsylvania from the NIH-NIAMS and Valiant for unrelated studies of atopic dermatitis. He was on an advisory board sponsored by Leo, which disbanded about 2 years ago, and is currently a consultant for Leo both with respect to atopic dermatitis. Dr Bunya has served as a consultant for Verily and receives grant funding from 10.13039/100007489Bausch and Lomb for an unrelated study. Dr Massaro-Giordano has served as a consultant for Verily and was on advisory boards sponsored by Dompe and Lynthera. Author Barbieri has no conflicts of interest to declare. The funding sources had no role in the design and conduct of the study; collection, management, analysis, and interpretation of the data; preparation, review, or approval of the manuscript; and decision to submit the manuscript for publication.
